# The Meaning of an Eight-Hour Day

**Published:** 1919-09-13

**Authors:** 


### THE MATRONS' AND SISTERS' DEPARTMENT.

### THE MEANING OF AN EIGHT-HOUR DAY.

It is clear from the schemes of reform which from time to time we are enabled to report that matrons and managers interpret the expression "an eight-hour day" very variously. It may indicate that the nurses put in an average of eight hours' work for seven days in the week, or fifty-six hours a week, with all kinds of extraneous duties, such as lecturing, correcting notes, writing reports, posting up books, and the like not reckoned as work, and with mealtimes snatched in the wards counted as time off. The eight-hour day, thus interpreted, becomes a doubtful blessing. Long before it was heard of the nurses in all well-managed institutions fared better than that.

On the other hand, the eight-hour day pushed to extremes becomes something of an absurdity. It has been taken to mean that nurses are to work only eight hours a day for six days in the week, that out of that eight hours one hour for meals is to be reckoned as "work," and that no attendance on lectures or classes is to be expected from the probationer unless it falls within her eight hours of duty. At this rate the number of hours put in by the probationer would not total more than forty in the week, and it is doubtful if she could acquire adequate knowledge of her business in the three years allotted to the course.

As generally formulated and gaining ground in the country, the eight-hour day means a forty-eight hour week, and implies that one day's rest in seven is allowed. Its adoption in institutions of various types has been enormously facilitated by the issue of the report of the Salaries Committee of the College of Nursing, and none who have the responsibility of advising managers in respect to the working hours of the nursing staff in institutions should fail to consult this able document. The scheme lately put forth by the Metropolitan Asylums Board is in accord with the valuable suggestions contained in this report. It is based on the same principle of a forty-eight-hour week, combined with a one day's rest in seven. All over the country Poor Law infirmaries are now adopting it, and voluntary hospitals, although much hampered by want of space and money to increase the nursing staff, are slowly coming into line.

There are two debatable points in settling the eight-hour day with precision. The first concerns the hours for meals and the second the hours for lectures. Asylum staffs have done good service in definitely insisting that all meals taken in wards or in the company of patients are to be reckoned among duty hours. Hospital nurses are never required to take their meals with patients, but night nurses at any rate are very commonly compelled to eat with one eye on the ward, and in convalescent homes and similar institutions the bad habit still prevails in places of gathering the whole household together for the midday meal. There can be no difference of opinion about the fact that it is not recreation to eat under such conditions. But this seems to us a very different thing from including among duty hours meals at which a sister or nurse presides as one among her equals. If to preside means to carve for a large number it must count as work. But this is rare under modern conditions, and meals rightly ordered constitute the most natural and agreeable form of recreation known to man or woman.

Again, one of the main objects of lessening the duty hours of probationers is to afford them more time for their studies. If they are not to attend lectures outside their daily eight hours of work, if they are not to keep the engagements of the class-room as the most important among those which they make for the weekly day off, then they are placed no longer in the category of pupils, but rather in the status of domestic helps, whose off-duty hours may be absorbed in amusement unrebuked. It would seem that some very zealous advocates of the nurses' cause forget that probationers are passing through one of the most critical periods of their lives while they are in hospital, and that the aim of lessening their daily spells of monotonous toil is to induce them to concentrate more successfully on the higher aspects of their calling. Hospital work, so far as it falls on the probationer, is not of a nature to exhaust her mind. It taxes her attention, her memory, her self-control, but it cannot be compared with that which will be demanded of her later on. To pass from bedmaking and cleaning and elementary nursing duties, after a period of refreshment in the open air, to the lecture and class-room is such a change of occupation as means actual recreation. Probationers, after all, are not children.

